# A Hybrid U-Shaped Deep Learning Network for Intracerebral Hemorrhage Segmentation in CT Scans

**DOI:** 10.3390/s26134164

**Published:** 2026-07-02

**Authors:** Ming Deng, Jiazuo Yao, Qingxiang Wu, Shihua Liang, Hailing Liang, Haihua Tang

**Affiliations:** 1 School of Computer Science and Engineering, Guilin University of Technology, Guilin 541006, China; gutming@glut.edu.cn (M.D.); gutzuo@glut.edu.cn (J.Y.); 2009009@glut.edu.cn (S.L.); 2 Department of Neurology, the 924th Hospital of the Joint Logisties Support Force of the Chinese People’s Liberation Army, Guilin 541000, China; 13877360072@163.com; 3 Hospital of Guangxi Normal University, Guilin 541004, China; lianghailing@gxnu.edu.cn; 4 Guangxi Key Laboratory of Embedded Technology and Intelligent System, Guilin University of Technology, Guilin 541006, China

**Keywords:** intracerebral hemorrhage, computed tomography, image segmentation, deep learning, Transformer, attention mechanism

## Abstract

Computed tomography (CT) scan is a widely used, non-invasive, sensor-based imaging technique that provides critical intracranial information for rapid stroke assessment. Accurate segmentation of intracerebral hemorrhage (ICH) in sensor-derived CT images is vital for clinical decision-making. Effective intelligent analysis of CT images is key to achieving reliable computer-aided diagnosis. However, existing deep learning methods struggle with complex ICH lesions characterized by blurred boundaries, irregular shapes, and large-scale variations. To address these challenges, this paper proposes TransAMGNet, a hybrid U-shaped network with Transformer integration for ICH CT image segmentation. The network is built on a residual U-Net backbone and introduces a Transformer encoder to strengthen global context modeling, thereby improving the representation of complex lesion morphology. Specifically, in the encoding stage, we design an Adaptive Dual-branch Channel Attention Module (ADCAM), which jointly models global and local channel information to enhance the model’s sensitivity to important feature responses. In the skip-connection pathway, we introduce a Multi-scale Feature Enhancement Module (MFEM), which preserves high-resolution spatial details while supplementing multi-scale contextual information to improve shallow-deep feature fusion. During decoding, a Gate-enhanced Dynamic Upsampling Module (GDUM) is constructed to improve the recovery of lesion boundaries and fine-grained structures through the synergy of gated recalibration and content-aware upsampling. The proposed method is systematically evaluated through comparative experiments and ablation studies. Experimental results show that TransAMGNet outperforms competing methods across multiple evaluation metrics, achieving Dice, Recall, IoU, Precision, and HD95 values of 90.47 ± 0.58%, 87.83 ± 3.71%, 81.26 ± 0.78%, 91.13 ± 0.95%, and 32.94 ± 1.1, respectively. The ablation studies further verify the effectiveness of each module. These results demonstrate that TransAMGNet can effectively improve segmentation performance for complex ICH lesions.

## 1. Introduction

Intracerebral hemorrhage (ICH) is an acute and high-risk subtype of stroke caused by spontaneous rupture of cerebral blood vessels, with high mortality and incidence rates [1]. Nearly 66% of global deaths from neurological disorders are associated with hemorrhagic stroke [2]. The overall global incidence of spontaneous intracerebral hemorrhage is 24.6 cases per 100,000 person-years [3]. The 30-day mortality rate ranges from 40% to 50%, and approximately half of the deaths occur within the first 24 h [4,5]. Because ICH has an abrupt onset and progresses rapidly, early and accurate assessment of the extent and location of hemorrhage is crucial for clinical intervention and prognosis estimation.

Computed tomography (CT), as a representative medical imaging sensing modality, has become one of the most important tools for the rapid diagnosis and assessment of ICH in clinical practice due to its fast acquisition, low cost, and high sensitivity to acute bleeding. In addition to conventional non-contrast CT (NCCT), computed tomography perfusion (CTP) imaging provides complementary hemodynamic information for ICH assessment. CTP can quantify perfusion-related parameters, such as cerebral blood flow, cerebral blood volume, mean transit time, and time-to-peak, thereby helping evaluate perihematomal perfusion abnormalities and potential secondary tissue injury. CTP-derived findings, including perihematomal hypoperfusion and dynamic contrast extravasation signs, can provide additional imaging markers for assessing the risk of hematoma expansion and unfavorable prognosis [6]. Therefore, CTP may play an auxiliary role in identifying patients at higher risk of hematoma growth and in understanding the pathophysiological progression of ICH.

However, CTP requires contrast administration, additional scanning protocols, and post-processing, and it is not routinely acquired for all ICH patients. In contrast, NCCT remains the most widely used first-line imaging modality for rapid ICH diagnosis, localization, and lesion quantification. Therefore, considering its clinical accessibility and routine use, this study focuses on automatic ICH segmentation based on NCCT images. In this study, unless otherwise specified, ICH CT images refer to NCCT images. Nevertheless, manual interpretation and lesion delineation on ICH CT images are time-consuming, labor-intensive, and highly dependent on physician experience. Meanwhile, ICH CT images often present blurred boundaries, heterogeneous intensity distributions, irregular lesion morphology, and substantial inter-patient scale variations, which make accurate hemorrhage segmentation challenging for conventional medical image processing methods. Therefore, automatic segmentation of ICH in CT images is of great practical significance for improving diagnostic efficiency, quantifying lesion extent, and supporting timely clinical decision-making.

In recent years, deep learning has advanced the development of medical image segmentation. In deep learning-based medical image segmentation, convolutional neural network (CNN) architectures represented by U-Net [7] have become the mainstream approach. Their encoder–decoder structure and skip-connection mechanism have shown strong capability in multi-scale feature fusion, which has further promoted the development of a series of improved models such as UNet++ [8], Res-UNet [9], Dense-UNet [10], MultiResUNet [11], and Double U-Net [12]. However, the convolutional kernel in CNNs is essentially a local operator. Owing to its limited receptive field, it is difficult for CNNs to model global contextual relationships adequately, which restricts their ability to handle ICH lesions with diverse morphology and broad spatial distribution.

To enhance global modeling capability, researchers have introduced Transformer [13] into medical image segmentation tasks. Benefiting from the advantage of self-attention in modeling long-range dependencies, Transformer can capture global semantic relationships over a larger spatial range [14], with representative examples including ViT [15] and Swin Transformer [16]. In medical segmentation, TransUNet [17] was the first to incorporate a Transformer into the U-Net encoder, effectively combining global semantics with local details, while 3D TransUNet [18] further demonstrated the great potential of this hybrid architecture for three-dimensional medical data. Subsequently, hybrid models such as UCTransNet [19], Swin-Unet [20], MedT [21], and TransBTS [22] further confirmed the feasibility of this direction. In general, the integration of U-shaped architectures with Transformer preserves the sensitivity of CNNs to local details while effectively strengthening global contextual modeling, which is important for boundary recognition and structural restoration.

However, existing medical image segmentation models still have some limitations when dealing with ICH CT images, mainly because the hemorrhage regions are often small, irregular in shape, and blurred at the boundaries [23]. To address this problem, we propose TransAMGNet, a U-shaped network for ICH CT image segmentation. The proposed model introduces targeted improvements at three key stages: encoding, skip connections, and decoding. In the encoder stage, we design an Adaptive Dual-branch Channel Attention Module (ADCAM), which enhances discriminative feature extraction by jointly integrating global and local information through dual-branch channel modeling. In the skip connection stage, we introduce a Multi-scale Feature Enhancement Module (MFEM), which preserves high-resolution spatial details while incorporating multi-scale contextual information, thereby improving the semantic representation and scale adaptability of skip features. In the decoder stage, we design a Gate-enhanced Dynamic Upsampling Module (GDUM), which improves the reconstruction of lesion boundaries and fine-grained structures through the joint effect of gated enhancement and dynamic upsampling.

In summary, the main contributions of this work are summarized as follows:

- We propose an Adaptive Dual-branch Channel Attention Module (ADCAM), which adaptively recalibrates feature responses by jointly modeling global and local channel descriptions, thereby improving the model’s ability to perceive key semantic information in hemorrhage regions.
- We design a Multi-scale Feature Enhancement Module (MFEM), which uses multi-scale dilated convolutions to model contextual information and enhance the discriminative ability and scale adaptability of skip features. This helps reduce the representation gap between shallow details and deep semantic features, and improves the fusion quality between encoder and decoder features.
- We propose a Gate-enhanced Dynamic Upsampling Module (GDUM), which first uses skip connection features to perform gated recalibration on the features to be upsampled, and then combines dynamic upsampling to achieve content-aware feature reconstruction. This improves the recovery of details in complex boundaries and small lesion regions.
- Based on ADCAM, MFEM, and GDUM, we develop a hybrid U-shaped network for ICH CT Image Segmentation, namely TransAMGNet. Quantitative experiments and visual comparison results show that the proposed method outperforms existing mainstream methods on the ICH CT dataset, demonstrating its effectiveness and superiority in ICH CT segmentation.

## 2. Related Work

### 2.1. Incorporating Attention Mechanisms into Convolutional Neural Networks

Since the introduction of the pioneering AlexNet model, the structural design and optimization strategies of deep convolutional neural networks have continued to evolve. Attention mechanisms have become an important means of improving feature representation by enabling networks to selectively emphasize informative responses and suppress redundant information. Among them, the Squeeze-and-Excitation (SE) module [24] is a representative channel attention method. It introduces a squeeze operation based on global average pooling and an excitation operation for channel-wise feature recalibration. By explicitly modeling channel importance, SE improves the discriminative ability of convolutional features. However, the global pooling operation compresses each feature map into a single descriptor, which may weaken local structural and texture information that is important for segmenting small target regions.

To further improve attention modeling, CBAM [25] sequentially combines channel attention and spatial attention. It uses both average-pooling and max-pooling descriptors to enhance feature selection from complementary perspectives, thereby improving the network’s focus on informative regions. Nevertheless, its sequential channel-spatial attention design still relies on global statistical descriptors in the channel attention branch and may introduce additional operations when embedded into segmentation networks. ECA-Net [26] provides a more efficient alternative by avoiding dimensionality reduction and using a one-dimensional convolution to capture local cross-channel dependencies. This design greatly reduces model complexity while maintaining effective channel interaction. However, ECA mainly depends on global average pooling to generate channel descriptors, and thus its ability to preserve local texture variations and fine-grained lesion structures remains limited.

Other studies have attempted to enhance attention modeling from different perspectives. GSoP [27] introduces second-order pooling to strengthen the modeling of statistical correlations among features. The GE module [28] employs depthwise separable convolution to expand the spatial receptive field and integrate contextual information efficiently. The scSE module [29] combines channel recalibration and spatial excitation, while GCNet [30], A2-Nets [31], and DAN [32] further exploit non-local or dual-attention mechanisms to capture long-range dependencies. Although these methods improve feature representation, they still involve trade-offs among global context modeling, local detail preservation, and computational complexity.

For intracranial hemorrhage CT segmentation, hemorrhagic regions are often small, boundary-blurred, and morphologically irregular. Therefore, attention modules should not only model channel dependencies efficiently but also preserve local texture and boundary-related information. The ADCAM proposed in this paper introduces a dual-branch channel attention strategy that combines global average pooling and local convolutional pooling. The global branch captures image-level contextual information, whereas the local branch enhances sensitivity to local structures and fine-grained hemorrhagic regions. Moreover, adaptive learnable fusion weights are used to balance the contribution of global and local channel descriptors. In this way, ADCAM improves segmentation performance while maintaining lightweight computational characteristics.

### 2.2. Skip Connections in U-Shaped Network Architectures

Skip connections in U-Net are intended to bridge the semantic gap between the encoder and the decoder and to recover fine-grained object details effectively [33,34,35]. UNet++ [8] introduces a Dense-like structure with nested and dense skip connections to enhance information flow across multi-scale features. U-Net3+ [36] incorporates a multi-scale feature aggregation strategy into skip connections and realizes sufficient cross-level interaction through full-scale skip connections. MultiResUNet [11] enhances skip connections through the introduction of the ResPath module and residual structures so as to address the semantic gap. Attention U-Net [37] embeds attention gates in skip connections, adaptively suppressing irrelevant background features while strengthening responses in lesion regions. RA-UNet [38] introduces a 3D hybrid residual attention-aware approach for precise feature extraction along skip connections. BCDU-Net [39] adds a bidirectional convolutional long short-term memory (LSTM) module to the skip-connection path.

These methods indicate that skip connections are not merely channels for information transfer, but key components that affect feature fusion quality and decoding performance. Especially in medical image segmentation tasks involving complex lesion morphology and significant scale variation, simply passing original shallow features directly is often insufficient to fully exploit the role of skip connections. How to enhance the semantic discriminability and multi-scale expressive power of skip features while preserving spatial details remains an issue worthy of further study.

### 2.3. Feature Upsampling

Feature upsampling is a key component in many U-shaped models and is mainly used to progressively restore the spatial resolution of feature maps. The most common upsampling methods include nearest-neighbor interpolation (NN) and bilinear interpolation, both of which reconstruct features based on fixed interpolation rules. These methods are computationally efficient but lack adaptivity.

To improve the representational capacity and flexibility of upsampling, some studies have introduced learnable upsampling mechanisms, such as transposed convolution (deconvolution) commonly used in instance segmentation and pixel shuffle [40] used in image super-resolution. However, these methods have certain limitations: transposed convolution is prone to checkerboard artifacts [41], whereas pixel shuffle performs inadequately in modeling complex structures and is therefore insufficient for advanced vision tasks.

With the rise of research on dynamic networks, an increasing number of dynamic upsampling modules have been proposed and have shown advantages across multiple tasks. CARAFE [42] performs content-aware reassembly by generating adaptive kernels for feature upsampling, enabling a larger effective receptive field and stronger local structure reconstruction than fixed interpolation. Subsequent methods such as FADE [43] and SAPA [44] further utilize high-resolution guidance features to generate dynamic kernels, thereby improving structure-aware feature reconstruction. However, kernel-based dynamic upsampling methods usually require additional kernel-generation networks and dynamic convolution operations, which may increase computational burden and implementation complexity. DySample [45] formulates dynamic upsampling from the perspective of point sampling rather than dynamic convolution. By learning sampling offsets, DySample enables content-adaptive feature resampling in a simple and flexible manner, avoiding the complex dynamic kernel generation process used in some kernel-based upsampling methods.

In U-shaped segmentation networks, the skip features contain rich spatial and boundary information, whereas the decoder features provide high-level semantic responses. Therefore, incorporating cross-level guidance before upsampling can help the decoder emphasize lesion-related responses and improve the recovery of boundary details and small hemorrhagic regions.

Based on this motivation, the proposed GDUM integrates gated feature enhancement with DySample-based dynamic upsampling. Specifically, the skip-connection features are used to guide residual gated recalibration of decoder features before upsampling, allowing hemorrhage-related semantic responses to be strengthened while preserving the original decoder representation. DySample is then employed to perform content-adaptive upsampling. Compared with directly using a standard upsampling operator in the decoder, GDUM further exploits the complementary relationship between encoder spatial details and decoder semantic information, which is beneficial for restoring fine-grained lesion structures and boundary details in ICH CT segmentation.

## 3. Methods

### 3.1. Overview of TransAMGNet

**Figure 1:**
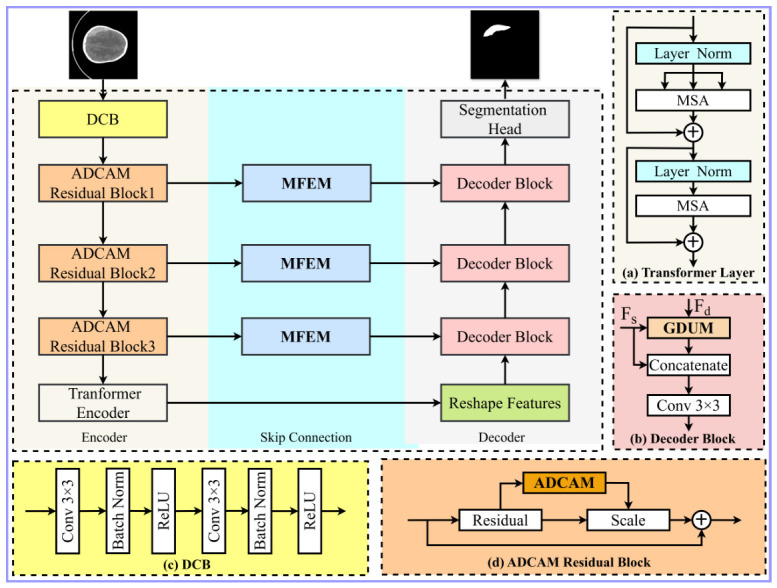
Illustration of the proposed TransAMGNet. (**a**) Structure of the Transformer layer, (**b**) Structure of the Decoder Block, (**c**) Double convolution blocks (DCB), and (**d**) Structure of the ADCAM Residual Block.

The input of a CT image can be denoted as $X\in R^{C\times H\times W}$, where C denotes the number of channels and H×W is the spatial resolution. Medical image segmentation aims to predict pixel-wise semantic labels with the same size as the input image. The proposed TransAMGNet follows a typical U-shaped encoder–decoder architecture, as illustrated in Figure 1.

**Encoder**: The input feature map $X\in R^{3\times 512\times 512}$ is converted into a feature map $M_{0}\in R^{64\times 512\times 512}$ using double convolution blocks as the input of the first encoder layer. Then, the three encoder layers are downsampled sequentially to obtain feature maps $M_{1}\in R^{128\times 256\times 256}$, $M_{2}\in R^{256\times 128\times 128}$, and $M_{3}\in R^{512\times 64\times 64}$ respectively. The classical U-Net encoder is built by stacking convolution and pooling layers. Although simple and intuitive, its manually designed feature extraction capacity is limited for complex medical imaging tasks such as ICH segmentation. To enhance the feature representation capability of the encoder, existing studies have proposed various U-Net variants, such as ResUNet [9] and Attention U-Net [37], which improve feature modeling ability by introducing deep residual networks, thereby enhancing the recognition ability of target regions.

In this study, we insert the proposed Adaptive Dual-branch Channel Attention Module (ADCAM) into the residual backbone of the CNN encoder. This design enhances the response to lesion-related features while keeping the computational overhead as low as possible, thus providing semantically rich inputs for subsequent processing. Next, similar to TransUnet, we employ a Transformer encoder consisting of 12 Transformer layers to model global contextual dependencies in the image, further enhancing the model’s capacity to capture global semantic information.

**Skip connections**: During the skip-connection process, the proposed MFEM is used to enhance the feature maps $M_{1}$, $M_{2}$, and $M_{3}$ extracted by the CNN encoder, producing $M_{1}^{\prime }$, $M_{2}^{\prime }$, and $M_{3}^{\prime }$ with the same channel numbers and spatial sizes. This enriches multi-scale contextual information and preserves key semantic details.

**Decoder**: The decoder consists of a series of decoder blocks and follows the standard U-Net upsampling strategy, where the channel number *C* is halved and the spatial resolution H and W are doubled. However, in the upsampling stage, we replace the conventional upsampling operation with Gate-enhanced Dynamic Upsampling Module (GDUM). GDUM can more effectively recover spatial details in structurally complex or boundary-blurred regions, compensating for the limitations of traditional upsampling and improving boundary reconstruction.

### 3.2. Adaptive Dual-Branch Channel Attention Module (ADCAM)

For channel attention, existing methods such as SE and ECA mainly rely on global descriptors to estimate channel importance. Although this design is efficient, it may overlook local texture variations and subtle boundary patterns that are critical for small hemorrhagic regions in ICH CT images.

**Figure 2:**
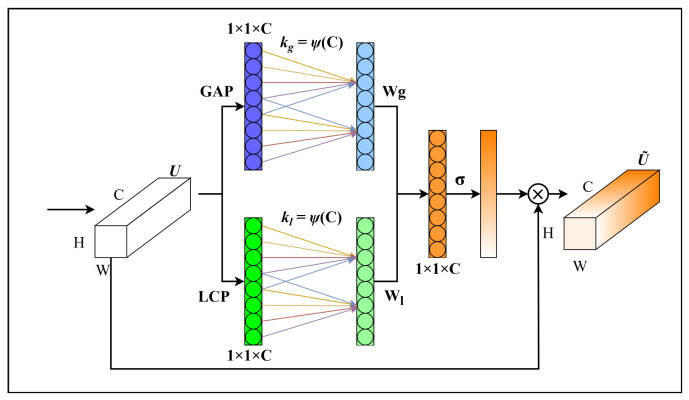
Structure of ADCAM.

To address this limitation, we design the Adaptive Dual-branch Channel Attention Module (ADCAM), as shown in Figure 2. The module uses separate global and local attention pathways to improve the model’s sensitivity to the morphology and boundary information of hemorrhagic regions.

We use global average pooling and local convolutional pooling, respectively, so that the network can effectively learn channel-wise spatial information and local regional information from the input feature $U\in R^{C\times H\times W}$. They are defined as follows:

(1) $$Z_{c}^{global}=P_{avg}\left(U\right)$$

(2) $$Z_{c}^{local}=P_{max}\left(DWConv\left(U\right)\right)$$

where $P_{avg}$ denotes Global Average Pooling, $P_{max}$ denotes Global Max Pooling, and DWConv denotes a depth-wise convolution with kernel size k = 3.

Then, one-dimensional convolutions with a specific kernel size k are used to capture cross-channel dependencies. In general, a small k is selected adaptively according to the number of channels. In this study, both $k_{g}$ and $k_{l}$ are set to 3. They are defined as follows:

(3) $$C_{g}=\mathrm{Conv}1D_{k_{g}}(Z_{c}^{global})$$

(4) $$C_{l}=\mathrm{Conv}1D_{k_{l}}(Z_{c}^{local})$$

Next, we introduce a pair of fixed learnable parameters, $W_{g}$ and $W_{l}$, for each channel and apply a softmax normalization to ensure that $W_{g}$, $W_{l}\in$ (0,1) and $W_{g}$ + $W_{l}$ = 1 (with $W_{g}$ = $W_{l}$ during initialization). This allows ADCAM to adaptively modulate the proportion of global and local information according to the feature distribution of each channel. The formulation is defined as follows:

(5) $$Z_{c}=C_{g}\cdot W_{g}+C_{l}\cdot W_{l}$$

Then, a Sigmoid function is then applied to compute the activation of the 1D convolution output, generating the weight vector $\omega \in R^{1\times 1\times c}$, which represents the local dependencies and importance of each feature channel. ω is given by:

(6) $$\omega =\mathrm{Sigmoid}(Z_{c})$$

Finally, to re-encode the features of each channel in *U*, the module performs element-wise multiplication between ω and *U* to obtain the weighted features $\tilde{U}$. Higher weights are assigned to important features to enhance them, while lower weights suppress irrelevant features through self-attention.

We integrated the ADCAM into each residual block (ResNet50 [34] was used in this study), as illustrated in Figure 1a. By recalibrating the channel features generated by the residual blocks, this module helps the network focus more effectively on important image regions, thereby enhancing the model’s representational capacity and ultimately improving its recognition performance.

### 3.3. Multi-Scale Feature Enhancement Module (MFEM)

In U-shaped segmentation networks, skip connections mainly transfer high-resolution spatial information from the encoder to the decoder to compensate for detail loss caused by downsampling. For ICH CT image segmentation, such shallow features are especially important for lesion boundary recovery. However, shallow skip features usually emphasize local edges and textures, while their semantic discriminability is relatively limited. When they participate in encoder–decoder fusion only in a single-scale form, they are often insufficient to represent the complex scale variation, morphology, and local context of ICH lesions. Therefore, this paper introduces the Multi-scale Feature Enhancement Module (MFEM) into the skip-connection pathway. While preserving the original spatial details, MFEM enriches skip features with multi-scale contextual information, thereby improving their discriminability and scale adaptability.

**Figure 3:**
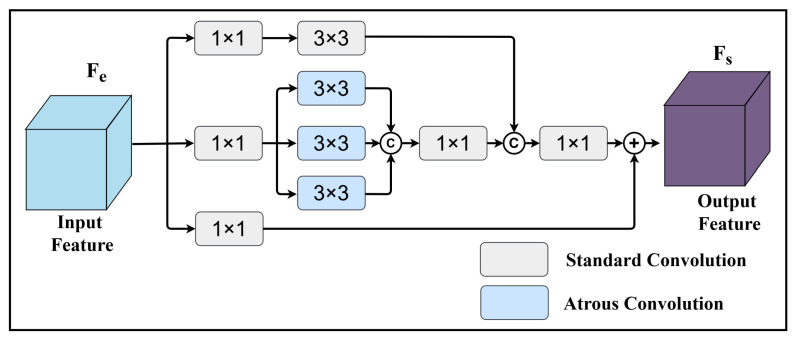
Structure of MFEM.

The overall structure of MFEM is shown in Figure 3. Specifically, MFEM adopts a parallel multi-branch structure to enhance the encoder skip feature $F_{e}\in R^{C\times H\times W}$. First, the local branch uses a 1×1 convolution for channel adjustment and then applies a 3×3 standard convolution to extract local structures and edge details. The formulation is defined as follows:

(7) $$F_{l}=Conv_{3\times 3}(Conv_{1\times 1}(F_{e}))$$

Then, the multi-scale context branch also first uses a 1×1 convolution for channel compression, and then applies three parallel 3×3 dilated convolutions with dilation rates of 1, 3, and 5 to extract contextual information under different receptive fields. The features from different scales are concatenated and fused by another 1×1 convolution. It is defined as follows:

(8) $$F_{m}=Conv_{1\times 1}(Concat(D_{1}(F_{e}^{\prime }),D_{3}(F_{e}^{\prime }),D_{5}(F_{e}^{\prime })))$$

where $Fe^{\prime }=Conv_{1\times 1}(F_{e})$, $D_{r}$(·) denotes a 3×3 dilated convolution with dilation rate *r*, and Concat(·) denotes channel-wise concatenation.

To avoid weakening the original high-resolution spatial information in the skip features during enhancement, MFEM also preserves an identity mapping branch and performs residual fusion with the enhanced multi-branch features. The final output is

(9) $$F_{s}=Conv_{1\times 1}(Concat(F_{l},F_{m}))+F_{e}^{\prime }$$

Lightweight in design, MFEM integrates both local and multi-scale contextual strategies, enabling the model to capture hemorrhage features across different scales. It provides enriched feature details for the decoder, improving adaptability to lesion boundaries and morphological variations, and particularly enhancing representation of small hemorrhages.

### 3.4. Gate-Enhanced Dynamic Upsampling Module (GDUM) in the Decoder Block

In the decoding stage of a U-shaped network, the spatial resolution of feature maps needs to be gradually restored and fused with the skip features from the encoder to achieve precise lesion localization and boundary reconstruction. For ICH CT images, hemorrhagic regions are often small, boundary-blurred, and morphologically irregular, which places higher demands on the recovery ability of the decoder. If traditional fixed upsampling strategies such as nearest-neighbor interpolation or bilinear interpolation are used directly, the implementation is simple and the computational cost is low, but the reconstruction process lacks adaptive modeling of local content. This can easily lead to the loss of high-frequency details and blurred boundaries, thereby reducing segmentation accuracy in small lesions and complex contour regions. Some learnable convolution-based upsampling methods provide better representation capacity, but they usually introduce extra parameters and computational cost, which is unfavorable for lightweight medical segmentation models [42].

**Figure 4:**
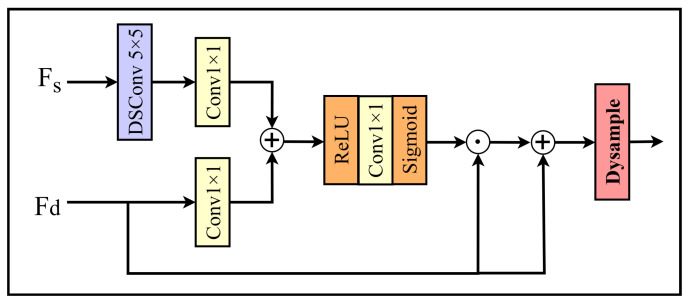
Structure of GDUM.

To address this problem, we design Gate-enhanced Dynamic Upsampling Module (GDUM), it combines gated enhancement with dynamic upsampling, as shown in Figure 4. Guided by the skip-connection features enhanced by MFEM, this module first performs gated recalibration on the decoder features to be upsampled so as to enhance responses related to target regions while suppressing irrelevant information. It then introduces DySample [45] as the dynamic upsampling method to improve recovery of complex boundaries and fine-grained structures. Finally, the decoder block fuses the upsampled features with the skip connection features so that high-level semantic information and high-resolution spatial details can be utilized together.

Specifically, the skip-connection feature map $F_{s}\in R^{C\times H\times W}$ from the current encoder level and the corresponding decoder feature map $F_{d}\in R^{2C\times \frac{H}{2}\times \frac{W}{2}}$ are used as the two branch inputs of the attention-gating mechanism. First, a 5×5 depthwise separable convolution is applied to adjust the resolution of $F_{s}$ to match that of $F_{d}$. Then, two parallel 1×1 convolutions are applied to $F_{s}$ and $F_{d}$, respectively, to generate the transformed features for gating:

(10) $$F_{S}=Conv_{1\times 1}(DSConv_{5\times 5}(F_{s}))$$

(11) $$F_{D}=Conv_{1\times 1}(F_{d})$$

These two features are then added point-wise and activated by ReLU, followed by another 1×1 convolution and a sigmoid activation to generate the feature weights:

(12) $$G=Sigmoid(Conv_{1\times 1}(ReLU(F_{S}+F_{D})))$$

A residual gating strategy is then used to recalibrate the decoder features:

(13) $$\tilde{F_{d}}=F_{d}+F_{d}⨀G$$

where ⨀ denotes element-wise multiplication. Residual gating preserves the original semantic information while strengthening useful responses, which helps prevent weak-response regions from being excessively suppressed and thus improves the preservation of small hemorrhages and blurred boundaries.

Next, DySample is used to upsample $\tilde{F_{d}}$, producing a high-resolution feature representation:

(14) $$F_{u}=DySample(\tilde{F_{d}})$$

**Figure 5:**
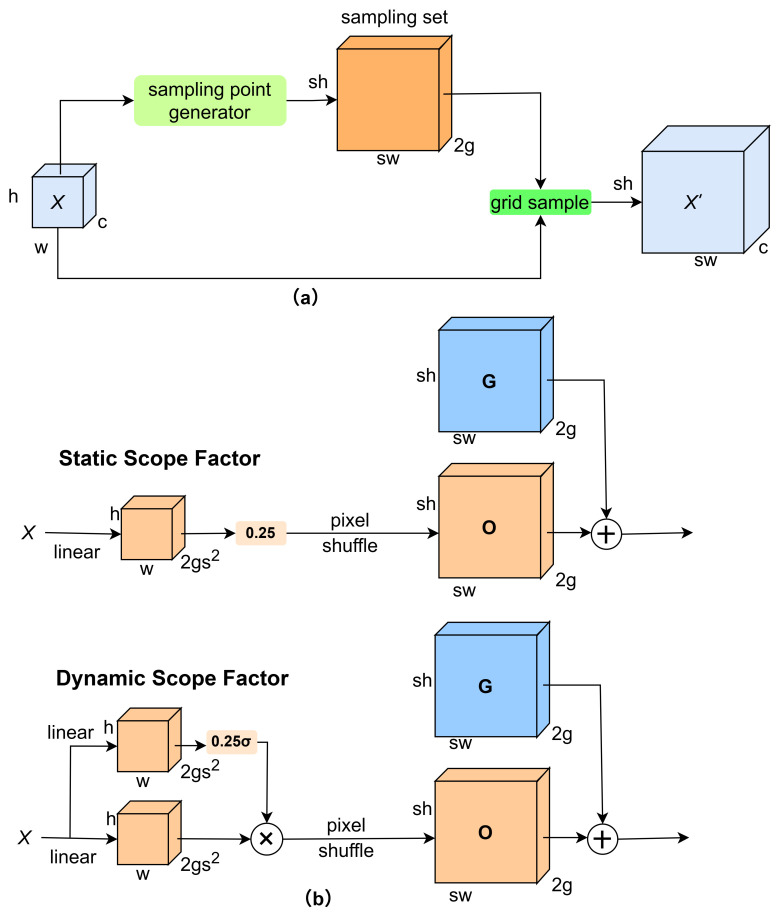
Structure of Dysample: (**a**) sampling-based dynamic upsampling; (**b**) Sampling point generator in DySample. The input feature, upsample feature, generated offset, and original grid are denoted by *X*, $X^{\prime }$, *G* and *O*, respectively. σ denotes the sigmoid function. sh represents the sampled height, sw represents the sampled width. $gs^{2}$ represents the number of channels after the feature graph passes through the linear layer.

As shown in Figure 5, as a lightweight dynamic upsampling method, DySample adaptively samples according to local feature content during the upsampling process, thereby improving reconstruction of complex structures and boundary details. Compared with fixed interpolation rules, DySample can recover spatial details more effectively without significantly increasing the computational burden.

Finally, the high-resolution feature map output by DySample is concatenated with the skip-connection feature map $F_{s}$, and a 3×3 convolution is applied for fusion to produce the output of the current decoder block:

(15) $$F_{out}=Conv_{3\times 3}(Concat(F_{u},F_{s}))$$

This design allows the decoder to preferentially enhance semantic responses related to hemorrhagic regions under the guidance of the gating mechanism and to further improve detail recovery through dynamic upsampling. The fusion with high-resolution skip features then enables more accurate representation of lesion boundaries and local structures.

### 3.5. Loss Function

During the training phase, TransAMGNet employs an end-to-end training strategy. We utilize the binary cross-entropy loss ($L_{BCE}$) and the Dice loss ($L_{Dice}$). The formulations for $L_{BCE}$ and $L_{Dice}$ are as follows:

(16) $$L_{BCE}=-\frac{1}{N}\sum_{i=1}^{n}\left(y_{i}\log\left(p_{i}\right)+\left(1-y_{i}\right)\log\left(1-p_{i}\right)\right)$$

(17) $$L_{Dice}=1-\frac{2\sum_{i=1}^{n}y_{i}p_{i}+\varepsilon }{\sum_{i=1}^{n}(y_{i}+p_{i})+\varepsilon }$$

(18) $$L_{Total}=\alpha \cdot L_{BCE}+\beta \cdot L_{Dice}$$

Here, n denotes the total number of pixels in each image; $y_{i}$ represents the ground truth value of the i-th pixel, and pi indicates the confidence score of the i-th pixel in the predicted result. In our experiments, the weighting coefficients are set as α = 0.55, β = 0.45, and the smoothing factor is ϵ = $10^{-6}$.

## 4. Experimental Setup

### 4.1. Dataset

#### 4.1.1. Public Datasets

**Figure 6:**
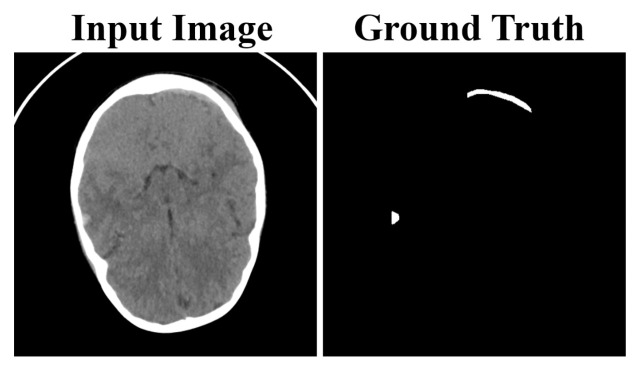
Examples of ICH CT images with corresponding semantic segmentation annotations.

The public ICH data used in this study were collected from multiple public sources, including the PHE-SICH-CT-IDS [46], BHSD [47], and CQ500 [48] datasets. We first extracted head CT images related to the study task together with their corresponding annotations from these datasets, and then screened them using unified quality-control criteria. Images with excessively low resolution, missing annotations, or severe artifacts that interfered with interpretation were removed. The retained samples were then reformatted and normalized in a unified manner to reduce organizational differences across data sources and to ensure consistency and comparability in subsequent experiments. In the end, 2560 original annotated two-dimensional CT slices with hemorrhagic regions were retained in the public dataset. These slices were adopted as the core foundation for model training and internal evaluation in this study, as shown in Figure 6.

#### 4.1.2. Guilin Hospital Dataset

**Figure 7:**
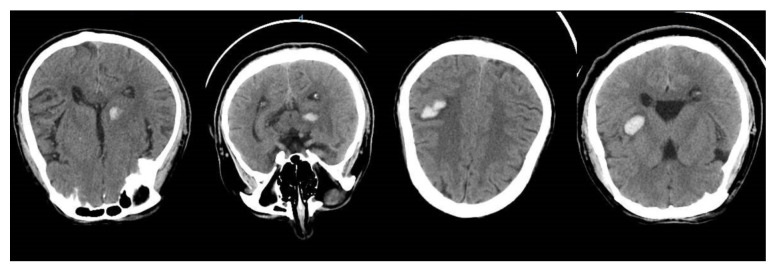
Example ICH CT images from the Guilin Hospital dataset.

To further evaluate the generalization performance of TransAMGNet on an independent clinical dataset, we used an independent clinical ICH CT dataset collected from a hospital in Guilin, referred to as the Guilin Hospital dataset. This dataset consisted of 77 original two-dimensional CT slices with hemorrhagic regions from 19 patients. The cohort included 13 male and 6 female patients, with a mean age of 53.37 ± 15.04 years and an age range of 10–87 years. All patients were clinically diagnosed with ICH, among whom 12 patients were diagnosed with hypertensive intracerebral hemorrhage. The hemorrhage locations included lobar hemorrhage, intraventricular hemorrhage, thalamic hemorrhage, and brainstem hemorrhage, with brainstem hemorrhage observed in 10 patients. Across the selected two-dimensional slices, the maximum in-plane lesion extent ranged from approximately 7 mm × 9 mm to 13 mm × 18 mm, and the CT attenuation value of the hemorrhagic regions was approximately 65 HU. As shown in Figure 7.

All CT images were manually and carefully annotated by professional radiologists. To protect patient privacy, all personally identifiable information was completely removed before analysis, and the dataset was anonymized in accordance with the ethical requirements of medical imaging research. Due to the retrospective and anonymized nature of the dataset, detailed prognostic information and standardized clinical severity scores, such as Glasgow Coma Scale, NIHSS, ICH score, modified Rankin Scale, and mortality outcomes, were not available in the records accessible for this study. Therefore, this study focuses on image-level hemorrhage segmentation performance rather than prognosis prediction or clinical severity stratification.

### 4.2. Data Preprocessing and Data Split

**Figure 8:**
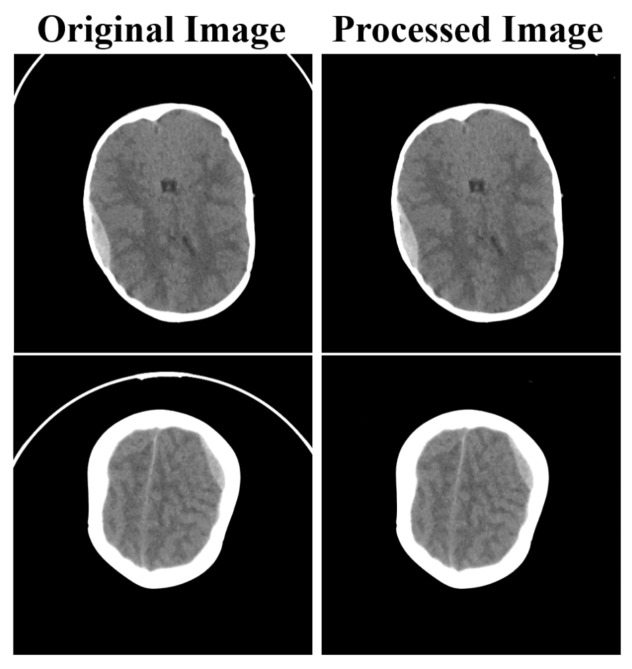
Removal of artifact regions in CT images.

During brain CT acquisition, obvious artifacts unrelated to the lesion may appear because of motion, metal, reconstruction errors, and other factors. These artifacts may increase training noise and interfere with the model’s effective learning of hemorrhagic regions. As shown in Figure 8, for the training set, we manually removed some obvious artifact regions from the images to reduce interference from non-lesion information. The validation and test sets were kept in their original form without such processing.

To avoid data leakage, all experiments in this paper adopt a patient-level split strategy. Specifically, for both the public dataset and the Guilin Hospital dataset, all two-dimensional CT slices from the same patient are assigned to only one subset among the training, validation, and test sets, without overlap across subsets. The target split ratio by slice number is 6:2:2 for the training, validation, and test sets.

**Figure 9:**
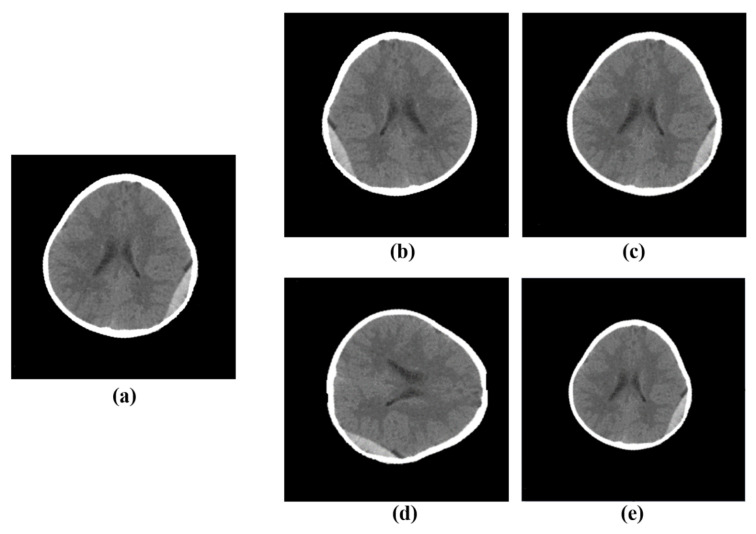
Examples of data augmentation: (**a**) original image; (**b**) horizontal flip; (**c**) random translation (10–20 pixels); (**d**) Random rotation by multiples of 90° (90°/180°/270°); (**e**) random scaling (70–90% of the original size).

In addition, to increase data diversity, we expanded the dataset through data augmentation, as shown in Figure 9. The augmentation process includes horizontal flipping, random translation, random rotation, and random scaling. After augmentation, the public dataset was expanded to 10,240 images, and the Guilin Hospital dataset was expanded to 308 images.

### 4.3. Evaluation Metrics

To evaluate the performance of the proposed TransAMGNet, we use several metrics, including the Dice coefficient, recall (i.e., pixel-level sensitivity), intersection over union (IoU), precision, and HD95. To further assess negative predictions in pixel-level segmentation tasks of ICH CT images, we also calculated the pixel-level specificity metric. Accordingly, Recall is equivalent to pixel-level Sensitivity. Except for HD95, the other evaluation metrics are related to four values: true positive (TP), true negative (TN), false positive (FP), and false negative (FN). These metrics are standard in computer vision and medical image segmentation and provide a multi-faceted evaluation of model accuracy, precision, and robustness. The formulations for these metrics are as follows:

(19) $$Dice=\frac{2TP}{2TP+FP+FN}$$

(20) $$Recall=Sensitivity=\frac{TP}{TP+FN}$$

(21) $$Specificity=\frac{TN}{TN+FP}$$

(22) $$IoU=\frac{TP}{TP+FP+FN}$$

(23) $$Precision=\frac{TP}{TP+FP}$$

(24) $$HD_{95}(G,P)=\max(prec_{95}\left\{\min_{p\in P}∥g-p∥|g\in G\right\},prec_{95}\left\{\min_{g\in G}∥p-g∥|p\in P\right\})$$

### 4.4. Implementation Details and Training Settings

To evaluate the robustness of the model to variations in data partitioning, the dataset was independently and randomly split five times using the same split ratio. For each split, the training, validation, and test sets were reconstructed, and the model was trained, validated, and tested independently. The final experimental results are reported as the mean ± standard deviation over the five independent experiments on the test set.

To ensure a fair comparison among different models, all compared methods were evaluated under consistent experimental settings, including the same dataset, preprocessing pipeline, training-set data augmentation strategy, loss function, model selection criterion, and evaluation metrics. Specifically, all models were trained and tested on the ICH CT dataset described in Section 4.1 and followed the data preprocessing strategy described in Section 4.2. The data augmentation operations applied to the training set included horizontal flipping, random translation, random rotation, and random scaling. All models were optimized using the loss function defined in in Section 3.5. Regarding the implementation of baseline methods, publicly available official implementations were preferentially adopted. For methods without official implementations, we re-implemented them in PyTorch 1.11.0 according to the original papers and kept their network architectures unchanged. For models with pre-trained backbone networks, the corresponding pre-trained weights were used to initialize the backbone networks, while layers without available pre-trained weights were randomly initialized. The optimal model weights were selected according to the validation performance and then evaluated on the corresponding test set.

All experiments were conducted on two NVIDIA GeForce RTX 4090 GPUs with 24 GB memory each. The batch size was set to 16, and the number of training epochs was set to 100. The initial learning rate was set to 0.001, and a polynomial learning-rate decay strategy with a power of 0.9 was adopted. Stochastic gradient descent was used as the optimizer, with a momentum of 0.9 and a weight decay of 0.001.

## 5. Experimental Results and Analysis

### 5.1. Comparison

#### 5.1.1. Analysis of the Comparison Results

To verify the effectiveness of the proposed TransAMGNet for ICH image segmentation, we compared it with several mainstream deep learning semantic segmentation methods in public dataset, including UNet [7], DeepLabV3+ [49], ViT [15], TransUNet [17], SETR [50], SwinT [16], SegFormer [51], U-2-Net [52], UCTransNet [19], and Swin-UNet [20]. All comparison methods were trained and tested under the same data split, preprocessing pipeline, and evaluation metrics. The relevant details have been described in Section 4.4.

As shown in Table 1, TransAMGNet achieved the best overall performance among all compared methods, with a Dice score of 90.47%, Recall/Sensitivity of 87.83%, pixel-level Specificity of 92.39%, IoU of 81.26%, Precision of 91.13%, and the lowest HD95 of 32.94. Compared with the second-best results, TransAMGNet improved Dice by 3.56%, Recall by 3.20%, pixel-level Specificity by 1.57%, IoU by 2.42%, and Precision by 3.34%, while reducing HD95 by 1.93. These results indicate that the proposed method not only improves the detection of hemorrhagic pixels but also better suppresses false-positive responses in non-hemorrhagic regions. Therefore, TransAMGNet achieves a more balanced segmentation performance in terms of lesion sensitivity, background specificity, region overlap, and boundary accuracy.

**Figure 10:**
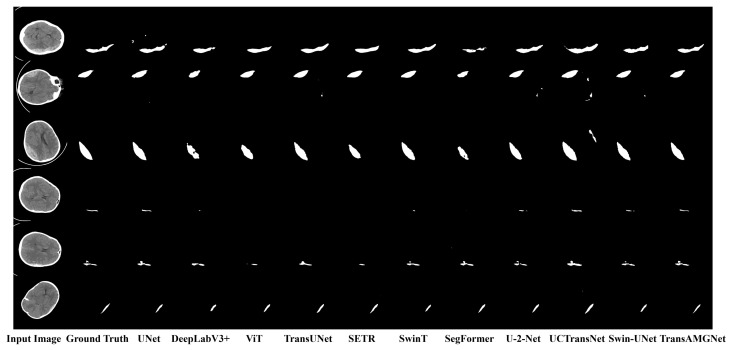
Visual comparison of segmentation results of bleeding areas by different methods; the first three rows compare large bleeding areas, and the last three rows compare small bleeding areas.

The visual segmentation results on the test set are shown in Figure 10. For large hemorrhagic regions, TransAMGNet can not only locate lesions accurately, but also recover their boundary morphology well. For small hemorrhagic regions, the proposed method also shows better segmentation ability, especially in the delineation of tiny lesion edges and preservation of structural integrity, where it outperforms most comparison models. This demonstrates that TransAMGNet has good robustness and fine-grained segmentation capability in complex scenarios.

#### 5.1.2. Evaluation of Significant Differences Between Compared Methods

To evaluate whether the differences among the compared methods were statistically significant, statistical analysis was performed based on the results of the five independent experiments from Table 1. For each evaluation metric, the Friedman test was first used to assess the overall differences among all methods, and Kendall’s W coefficient was calculated to estimate the consistency of method rankings across repeated experiments. Considering the limited number of independent repeated experiments and the difficulty of reliably validating the normality assumption, we used two-sided exact paired sign-flip permutation tests, followed by the application of the Holm–Bonferroni correction in each metric to control for multiple comparisons.

The results of the Friedman test and Kendall’s W coefficient indicate that there are significant overall differences in all indicators, including Dice (*p* = 0.001, W = 0.7753), Recall (*p* = 0.0024, W = 0.5455), Specificity (*p* = 0.001, W = 0.8785), IoU (*p* = 0.001, W = 0.9287), Precision (*p* = 0.001, W = 0.9676), and HD95 (*p* = 0.001, W = 0.8560). These results indicate that the compared segmentation methods exhibited statistically distinguishable overall performance patterns, with generally high ranking consistency across repeated experiments.

After performing paired exact sign-flip permutation tests between TransAMGNet and each competing method, Paired exact sign-flipping permutation tests were performed between TransAMGNet and each competing method. Under one-sided testing, nearly all uncorrected p-values reached 0.0312, indicating a consistent directional advantage of TransAMGNet. However, when the more conservative two-sided exact test was applied, the minimum achievable p-value was 0.0625. Following Holm–Bonferroni correction within each metric, none of the comparisons remained statistically significant (all p-values equal to 0.3125). Therefore, given the limited number of independent experiments, the statistical evidence of a significant advantage derived from these results should be interpreted with caution.

### 5.2. Experimental Results on the Guilin Hospital Dataset

To further evaluate the generalization ability and robustness of the model, we conducted experiments on the Guilin Hospital intracerebral hemorrhage CT image dataset. The dataset was split using the same preprocessing strategy in Section 4.2.

Two experimental settings were considered on the Guilin Hospital dataset. In the first setting, the model was trained only on the public dataset and then directly evaluated on the test set of the Guilin Hospital dataset, without using any in-hospital samples during training and without parameter updating or fine-tuning. This setting was used to assess the external generalization ability of the model on an independent clinical source. In the second setting, while keeping the test set independent and unchanged, the training subset of Guilin Hospital dataset was merged into the training set of the public dataset, and the model was retrained and evaluated on the same test set. This setting was used to analyze the change in model performance after introducing target-site samples and serves as a supplementary training experiment.

To further evaluate whether the performance difference between the two settings was statistically significant, we conducted significance analysis using the paired exact sign-flip permutation test. A one-sided p-value was reported under the pre-specified directional hypothesis that Setting 2 would outperform Setting 1, and Holm–Bonferroni correction was further applied to account for multiple comparisons.

As shown in Table 2, even without using in-hospital samples for training, TransAMGNet still achieved good segmentation performance on the test set of the Guilin Hospital dataset, indicating a certain degree of cross-source generalization capability. Meanwhile, compared with Setting 1, Setting 2 showed positive average improvements across all evaluation metrics. Among them, the Dice score reached the 0.05 significance level in the unadjusted one-sided paired exact test (*p* = 0.0313). However, after Holm–Bonferroni correction for multiple comparisons, none of the metrics reached the 0.05 significance level. Therefore, the observed performance gains should be interpreted as a positive and consistent improvement trend rather than conclusive evidence with sufficient statistical significance. This may be attributed to the limited dataset size, the small number of datasets splits, and the insufficient statistical power under small-sample conditions.

### 5.3. Inference Time

**Figure 11:**
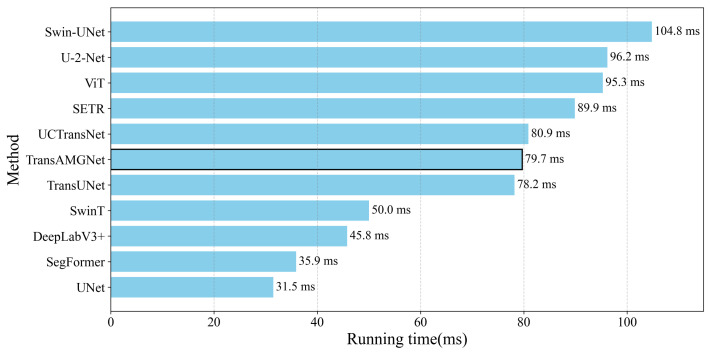
Average inference time of different models on the test set.

In order to more comprehensively evaluate the actual efficiency of the proposed model, we compared the average inference time of different models on the test set. The hardware environment used for testing is the same as that described in Section 4.4 to ensure comparability. As shown in the Figure 11, Swin-Unet has the longest inference time, while the traditional U-Net has the shortest. Although TransAMGNet is not the fastest model, its segmentation performance on all datasets is better than that of the other comparison methods. It therefore achieves higher accuracy while maintaining relatively short inference time.

Overall, these additional computational costs are reasonable and worthwhile because they effectively improve segmentation accuracy and lesion reconstruction quality. The proposed model achieves a good balance between high performance and efficiency, offering both accuracy and practical usability.

## 6. Ablation Study

### 6.1. Effect of Different Components of TransAMGNet

To understand the effect of different components of the proposed method and further validate the effectiveness of the proposed method, the experimental design is shown in Table 3. Specifically as follows:

(a) The attention mechanism is designed into two types: no mechanism is applied, ADCAM is used; (b) To apply or not to apply the Transformer encoder; (c) Skip connections include without and with MFEM; (d) The upsampling methods include three types: bilinear interpolation, deconvolution, and GDUM.

#### 6.1.1. Ablation Result Analysis

The experimental results are shown in Table 4. By comparing M1 and M6, we can see that introducing channel attention in the feature downsampling stage effectively improves feature extraction. Specifically, after ADCAM is embedded into the encoder, M6 shows clear performance gains over M1 while increasing the number of parameters by only 0.89M. Dice, Recall, IoU, and Precision improve by 5.09%, 5.7%, 4.44%, 4.28%, and 3.88%, respectively, and HD95 decreases by 1.99.

These results indicate that the introduction of the attention mechanism effectively strengthens the model’s ability to model key channel features, allowing discriminative semantic information to be preserved during feature downsampling and thereby improving segmentation quality. While keeping the network lightweight, ADCAM enhances the model’s perception of ICH lesions by introducing local contextual information and adaptively fusing it, which verifies the applicability and effectiveness of this mechanism in medical image segmentation.

By comparing M2 and M6, we can see that adding MFEM to the skip connections also improves all metrics, indicating that MFEM effectively enhances the features extracted by the CNN encoder during skip fusion. At the same time, the comparison between M3 and M6 shows that the Transformer encoder also contributes to the improvement in model performance.

**Figure 12:**
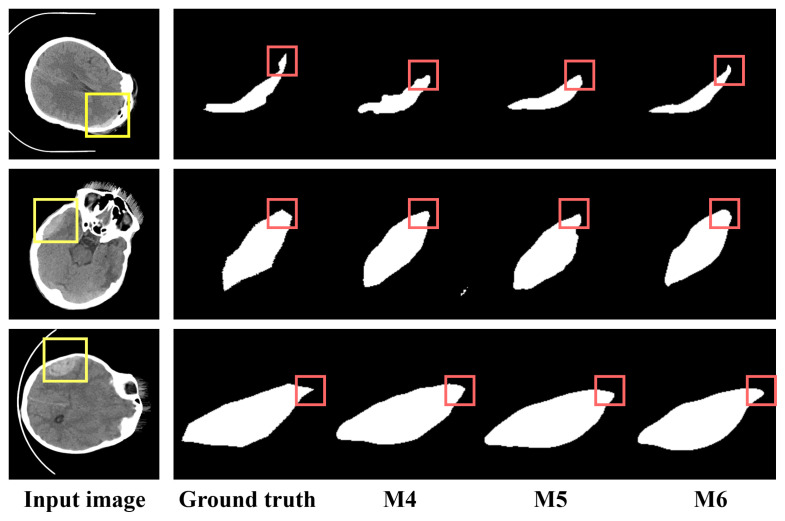
Comparison of M4, M5, and M6 in terms of edge details of the segmented area.

In addition, comparisons among M4, M5, and M6 show that in the upsampling stage, the GDUM-based setting achieves the best performance on all five metrics. Compared with bilinear interpolation (M4), it improves by 1.46%, 1.56%, 1.60%, 0.70%, and 1.18% on the first four metrics, respectively; compared with transposed convolution (M5), it improves by 1.13%, 1.87%, 0.86%, 0.45%, and 0.56%, respectively. It also achieves the best HD95 value of 32.94. These results indicate that GDUM can reconstruct lesion regions more accurately, especially in terms of boundary delineation. The visualization results in Figure 12 further show that M6 recovers boundary details better for CT images with different edge characteristics and has better ability to represent fine lesion boundaries, making it suitable for ICH images with complex structures, blurred boundaries, and clear regional variation.

#### 6.1.2. Significance Analysis of Ablation Results

The statistical analysis methods used for the results in Table 4 is consistent with the evaluation methods used for Table 1 in Section 5.1.2, except that parameters and FLOPs are regarded as deterministic indicators of model complexity and are not included in statistical tests.

For Table 4, the Friedman test also indicated significant overall differences among the experimental schemes for Dice (*p* = 0.0209, W = 0.4971), Recall (*p* = 0.0020, W = 0.6943), Specificity (*p* = 0.0010, W = 0.7371), IoU (*p* < 0.001, W = 0.9200), Precision (*p* < 0.001, W = 0.9829), and HD95 (*p* = 0.0010, W = 0.7763). The Kendall’s W values suggest moderate-to-high ranking concordance, especially for IoU, Precision, HD95, and Specificity, supporting the stability of the observed performance trends among different experimental schemes.

However, the statistical tests showed that these improvements did not reach the 0.05 significance level after Holm–Bonferroni correction. In particular, although several comparisons yielded an unadjusted one-sided exact *p*-value of 0.0312, the corresponding two-sided exact *p*-value was 0.0625, which did not meet the conventional 0.05 threshold. These results suggest that the ablation study provides evidence of consistent performance improvement trends rather than conclusive statistical significance for every incremental component, possibly due to the limited number of independent repeated experiments.

### 6.2. Ablation Study of ADCAM

#### 6.2.1. Results and Analysis of the Ablation Study

To further validate the effectiveness of the proposed ADCAM, we conducted an ablation study by progressively enabling the global branch, local branch, dual-branch structure, and adaptive fusion mechanism. As shown in Table 5, both the global-only and local-only variants improve the segmentation performance compared with the baseline without ADCAM, indicating that each branch contributes useful complementary information. Specifically, the global branch improves the Dice score from 85.38% to 86.93% and the IoU from 76.98% to 79.08%, suggesting that global contextual modeling helps enhance region-level consistency. The local branch achieves a Dice score of 87.11% and reduces HD95 from 34.93 to 33.82, indicating its advantage in capturing fine-grained local details and boundary information.

When the global and local branches are jointly used without adaptive fusion, the Dice score further increases to 89.52%, outperforming both single-branch variants. This demonstrates that the dual-branch design can effectively combine global contextual cues and local detail-sensitive representations, which is particularly beneficial for segmenting small hemorrhagic regions with weak contrast and irregular shapes. After introducing the adaptive fusion strategy, the full ADCAM achieves the best overall performance, with a Dice score of 90.47%, Recall of 87.83%, IoU of 81.26%, Precision of 91.13%, and the lowest HD95 of 32.94. These results indicate that adaptive fusion further enhances the interaction between the two branches and allows the network to dynamically emphasize the most informative features for accurate ICH segmentation.

#### 6.2.2. Variation in Channel Weights in ADCAM

To better understand the behavior of the channel attention mechanism during training, we analyzed the change in the difference ΔW between the global weight $W_{g}$ and the local weight $W_{l}$.

**Figure 13:**
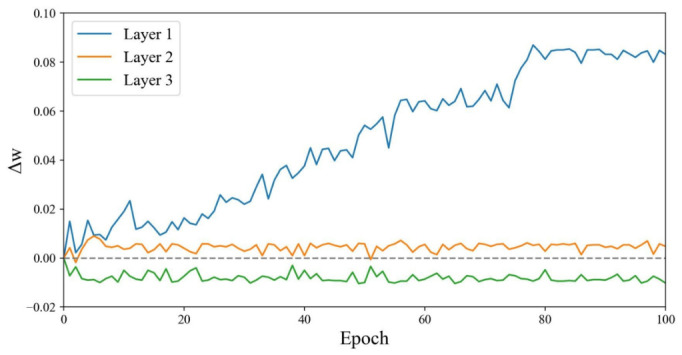
Variation of the difference ΔW = $W_{g}-W_{l}$ between the global weight $W_{g}$ and the local weight $W_{l}$ during training. A positive ΔW indicates that $W_{g}$ contributes more than $W_{l}$, and a negative ΔW indicates a greater contribution from $W_{l}$.

From Figure 13, we can see that the ΔW of the first-layer encoder increases continuously with training, indicating its exceptional sensitivity to global channel information and its tendency to integrate overall semantics in the early stages. The ΔW of the second-layer encoder is consistently slightly greater than 0, suggesting that it also pays attention to global information, but its dependence on global features is weaker than that of the first layer. In contrast, the ΔW of the third-layer encoder is slightly less than 0, implying a greater emphasis on local channel features. Overall, shallow features rely more on global semantics to build stable basic representations, while deep features gradually shift towards capturing and enhancing local details, thus achieving a feature modeling process from macro to micro.

### 6.3. Effect of Different Kernels and Dilation Rates in MFEM

We also conducted another set of experiments on the ICH dataset to evaluate the impact of different kernel size combinations and dilation rate configurations on model performance in the second branch of MFEM. Table 6 presents the experimental results, showing that different combinations of kernel size and dilation rate lead to differences. When the kernel combination includes larger kernels such as 5×5 or 7×7, the overall model performance improves, but relying solely on large kernels like 7×7 leads to a performance decrease. In addition, different dilation rate combinations also cause performance fluctuations. Based on these empirical observations, we choose [3, 3, 3] kernels and a dilation rate of (1, 3, 5) in all our experiments.

### 6.4. Ablation Study of Loss Functions with Different Weight Configurations

To justify the weighting configuration of the proposed loss function, we conducted an analysis using different loss formulations and weighting settings. As shown in Table 7, using BCE Loss or Dice Loss alone resulted in inferior overall performance compared with the combined BCE + Dice Loss. BCE Loss alone obtained the lowest Dice score of 83.15% and the highest HD95 of 42.86, while Dice Loss alone improved Recall but produced lower Precision, indicating that neither pixel-wise classification supervision nor region-overlap optimization alone was sufficient for accurate ICH segmentation.

Compared with Tversky Loss and Focal Loss, the combined BCE + Dice Loss achieved more competitive performance, suggesting its effectiveness in handling foreground-background imbalance and lesions with varying sizes. Among the tested weighting configurations, α=0.55 and β=0.45 achieved the best overall results, with a Dice score of 90.47%, IoU of 81.26%, Precision of 91.13%, and the lowest HD95 of 32.94. Although the configuration of α=0.50 and β=0.50 achieved a slightly higher Recall, the selected setting provided better overall segmentation accuracy and boundary stability. These results indicate that α=0.55 and β=0.45 offer a more balanced trade-off between lesion sensitivity, false-positive suppression, region overlap, and boundary accuracy.

## 7. Discussion

From the perspective of sensor-acquired medical imaging, CT scans provide fast and reliable structural information for emergency diagnosis, but the resulting images often contain complex lesion appearances and subtle boundary variations. For ICH segmentation, such characteristics increase the difficulty of accurately identifying hemorrhagic regions and preserving fine structural details, making robust feature extraction and boundary recovery particularly important for downstream computer-aided analysis.

To address these challenges, this paper proposes TransAMGNet for ICH CT image segmentation and designs it specifically around three key stages: encoding, skip connection, and decoding. Experimental results show that the model achieves better overall performance on Dice, IoU, Recall, Precision, and HD95, indicating clear advantages in lesion recognition, boundary recovery, and fine-structure reconstruction.

Specifically, ADCAM jointly models global and local channel information and adaptively recalibrates feature responses, which helps improve the model’s discriminative ability for small hemorrhages, blurred boundaries, and irregular lesions. MFEM preserves the high-resolution spatial details of skip connections while introducing multi-scale contextual information, thereby strengthening the semantic representation of shallow features and improving encoder–decoder fusion quality. GDUM combines gated recalibration and dynamic upsampling to improve the recovery of complex boundaries and local details in the decoding stage. The visualization results also show that TransAMGNet produces relatively complete segmentation with clearer boundaries for both large and small lesions. Because accurate delineation directly affects the extraction of clinically meaningful information, such as hemorrhage extent, morphology, and spatial distribution, these characteristics are particularly important for the analysis of sensor-acquired CT images.

Nevertheless, this study still has several limitations. First, a small number of images with obvious artifacts were manually processed during training, indicating that the current workflow still has some dependence on data quality control. Second, this work mainly adopts channel attention and does not explicitly incorporate spatial attention, so there remains room to improve the modeling of lesion spatial distribution and boundary localization. Third, the model relies on a Transformer encoder to model long-range dependencies, and this component accounts for a considerable proportion of the overall parameters and computational cost, which may be unfavorable for deployment in resource-constrained scenarios.

Additionally, the external validation dataset from Guilin Hospital included in this study is limited by its small sample size and single-center origin. Moreover, all evaluated CT slices contained visible hemorrhagic regions. Therefore, although pixel-level Specificity could be calculated based on non-hemorrhagic pixels within the annotated slices, image-level Specificity and false-positive behavior on bleeding-negative CT slices could not be fully evaluated. This may limit the assessment of the model’s robustness in real clinical screening scenarios where both hemorrhagic and non-hemorrhagic CT images are encountered. Meanwhile, although the inclusion of target-domain training samples led to positive average improvements across multiple evaluation metrics, these gains did not remain statistically significant after multiple-comparison correction. This may be partly attributed to the relatively limited size of the in-hospital dataset, the small number of independent repeat experiments, and the insufficient statistical power under small-sample conditions. Therefore, further thorough validation on larger, multi-center datasets containing both hemorrhage-positive and hemorrhage-negative CT images is warranted to more reliably assess the generalizability, image-level specificity, and clinical robustness of the proposed model.

## 8. Conclusions

To address the challenge of hemorrhage areas having fuzzy boundaries, irregular shapes, and large-scale variations in complex intracerebral hemorrhage CT image segmentation tasks, this paper proposes TransAMGNet, a U-shaped network that combines a Transformer encoder with collaborative multi-module enhancement. The model introduces the Adaptive Dual-branch Channel Attention Module (ADCAM), the Multi-scale Feature Enhancement Module (MFEM), and the Gate-guided Dynamic Upsampling Module (GDUM) into the encoding, skip-connection, and decoding stages, respectively, thereby jointly optimizing discriminative feature extraction, skip-feature enhancement, and lesion-detail recovery.

Experimental results show that TransAMGNet achieves good performance on multiple evaluation metrics on the public dataset and demonstrates a certain degree of external generalization on the Guilin Hospital dataset. This indicates that the proposed architecture can balance global context modeling, local detail preservation, and complex boundary reconstruction effectively, and is therefore suited to the ICH segmentation task.

Despite demonstrating promising segmentation performance on sensor-acquired CT images, the proposed method still has several non-negligible limitations: the Transformer encoder incurs substantial computational burden, the model relies solely on channel attention mechanisms without spatial attention complementation, and external validation is conducted on a limited dataset scale. Future work will focus on model lightweighting, multi-dimensional attention fusion, and other categories of medical image segmentation to further improve robustness, generalization ability, and clinical applicability.

## Figures and Tables

**Table 1:** The experimental segmentation results (Mean (%)) Standard Deviation) evaluated on the ICH dataset compared with different methods. The best results are highlighted in bold.

Methods	Dice (%)	Recall (%)	Specificity (%)	IoU (%)	Precision (%)	HD95
UNet [7]	77.37 ± 2.23	77.02 ± 7.07	81.51 ± 3.94	69.43 ± 1.44	80.99 ± 1.13	41.64 ± 3.49
DeepLabV3+ [49]	78.43 ± 5.06	76.96 ± 1.88	82.57 ± 3.27	77.16 ± 2.27	85.60 ± 1.07	36.75 ± 2.57
ViT [15]	84.39 ± 3.67	81.99 ± 6.28	88.94 ± 1.47	75.59 ± 1.13	84.62 ± 0.97	38.93 ± 2.16
TransUNet [17]	85.38 ± 2.89	82.13 ± 3.87	87.95 ± 3.64	76.98 ± 1.23	87.25 ± 0.99	34.93 ± 1.30
SETR [50]	83.53 ± 2.47	81.46 ± 3.38	86.42 ± 2.86	72.86 ± 0.46	86.83 ± 0.87	37.57 ± 1.51
SwinT [16]	86.91 ± 1.04	83.43 ± 4.64	90.82 ± 3.15	78.84 ± 0.83	86.97 ± 1.02	36.80 ± 2.04
SegFormer [51]	83.58 ± 3.44	82.94 ± 1.67	86.54 ± 2.65	71.63 ± 1.34	86.66 ± 1.08	35.56 ± 1.32
U-2-Net [52]	80.10 ± 1.17	79.51 ± 5.18	84.11 ± 2.11	78.50 ± 1.92	80.40 ± 1.11	39.46 ± 3.97
UCTransNet [19]	84.01 ± 5.72	80.30 ± 3.24	87.20 ± 3.52	75.38 ± 2.86	82.98 ± 1.76	42.67 ± 2.17
Swin-UNet [20]	85.95 ± 2.32	84.63 ± 0.24	89.31 ± 3.71	78.08 ± 1.71	87.79 ± 0.64	34.87 ± 1.05
**TransAMGNet**	**90.47 ± 0.58**	**87.83 ± 3.71**	**92.39 ± 1.61**	**81.26 ± 0.78**	**91.13 ± 0.95**	**32.94 ± 1.10**

**Table 2:** Test results on the Guilin Hospital dataset.

Metric	Setting 1	Setting 2	Mean Improvement	95% CI of Paired Improvement	One-Sided Exact *p*	Holm-Adjusted *p*
Dice (%)	88.14 ± 1.57	89.05 ± 1.82	0.908	[0.267, 1.549]	0.0312	0.1875
Recall (%)	87.59 ± 1.63	88.43 ± 1.34	0.834	[−0.332, 2.000]	0.0625	0.3125
Specificity (%)	91.37 ± 2.21	93.12 ± 1.19	1.150	[−0.728, 3.028]	0.0625	0.3125
IoU (%)	82.07 ± 1.59	82.96 ± 1.31	0.894	[−0.091, 1.879]	0.0625	0.3125
Precision (%)	88.92 ± 0.41	89.78 ± 0.86	0.860	[0.036, 1.684]	0.0625	0.3125
HD95	34.32 ± 1.15	33.26 ± 1.07	1.064	[0.191, 1.937]	0.0625	0.3125

**Table 3:** Ablation study design schemes.

	Design of Channel Attention Mechanism in Downsampling		Module in Skip Connections		Transformer Encoder		Upsampling Method		
Model	Not Applied	ADCAM	Not Applied	MFEM	Not Applied	Be Applied	Bilinear Upsampling	Transposed Convolution	GDUM
M0	✔		✔			✔	✔		
M1	✔			✔		✔			✔
M2		✔	✔			✔			✔
M3		✔		✔	✔				✔
M4		✔		✔		✔	✔		
M5		✔		✔		✔		✔	
M6		✔		✔		✔			✔

**Table 4:** Performance of different experimental scheme. The results of the method proposed in this study are shown in bold.

Method	Dice (%)	Recall (%)	Specificity (%)	IoU (%)	Precision (%)	HD95	Param (M)	FLOPs (G)
M0	85.38 ± 0.89	82.13 ± 3.87	87.95 ± 3.64	76.98 ± 1.23	87.25 ± 0.99	34.93 ± 1.30	105.91	56.8
M1	86.16 ± 0.61	84.17 ± 1.49	88.61 ± 2.44	78.22 ± 1.42	88.78 ± 1.10	33.54 ± 1.23	107.78	58.7
M2	88.51 ± 2.38	85.04 ± 2.98	90.07 ± 2.51	79.88 ± 0.94	89.36 ± 0.48	33.18 ± 1.27	107.63	58.2
M3	87.71 ± 2.28	84.23 ± 2.72	89.92 ± 2.18	78.66 ± 1.37	88.21 ± 0.98	35.42 ± 1.16	23.62	35.6
M4	89.01 ± 1.45	86.27 ± 2.25	90.79 ± 1.92	80.56 ± 0.64	89.95 ± 0.25	34.19 ± 1.19	107.84	59.4
M5	89.34 ± 4.65	85.96 ± 1.04	91.53 ± 2.33	80.81 ± 0.73	90.57 ± 0.39	34.27 ± 1.35	116.42	86.9
M6	**90.47 ± 0.58**	**87.83 ± 3.71**	**92.39 ± 1.61**	**81.26 ± 0.78**	**91.13 ± 0.95**	**32.94 ± 1.10**	**108.67**	**59.6**

**Table 5:** Performance of different ablation experimental scheme. The best results are shown in bold.

Variant	Global Branch	Local Branch	Adaptive Fusion	Dice (%)	Recall (%)	IoU (%)	Precision (%)	HD95
Baseline w/o ADCAM	✗	✗	✗	85.38 ± 0.89	82.13 ± 3.87	76.98 ± 1.23	87.25 ± 0.99	34.93 ± 1.30
Global-only branch	✔	✗	✗	86.93 ± 1.51	84.21 ± 2.15	79.08 ± 1.37	89.33 ± 0.83	34.45 ± 2.42
Local-only branch	✗	✔	✗	87.11 ± 1.36	83.47 ± 4.23	78.44 ± 0.95	88.91 ± 1.14	33.82 ± 1.51
Dual branch w/o adaptive fusion	✔	✔	✗	89.52 ± 0.73	86.92 ± 3.36	80.07 ± 1.18	90.85 ± 1.49	33.26 ± 1.23
Full ADCAM	✔	✔	✔	**90.47 ± 0.58**	**87.83 ± 3.71**	**81.26 ± 0.78**	**91.13 ± 0.95**	**32.94 ± 1.10**

**Table 6:** Comparison of Average Dice scores under different kernel sizes and dilation rates (Dilation Rate 1 (1,1,1), Dilation Rate 2 (1,3,5), Dilation Rate 3 (2,3,5)), with corresponding parameter counts. The best results are highlighted in bold.

Conv. Kernels	Param (M)	Dilation Rates 1	Dilation Rates 2	Dilation Rates 3
[1,3,5]	1.17	84.25	88.93	89.17
[1,3,7]	1.57	86.11	87.35	87.01
[1,5,7]	1.83	87.79	88.47	88.21
[3,3,3]	**1.04**	N/A	**90.47**	90.13
[3,5,7]	1.96	85.56	88.04	88.24
[5,5,5]	1.83	N/A	85.11	85.33
[7,7,7]	3.01	N/A	76.17	77.03

**Table 7:** Performance of different weight configurations. The best results are shown in bold.

Loss Function	Configuration	Dice (%)	Recall (%)	IoU (%)	Precision (%)	HD95
Tversky Loss	α = 0.3, β = 0.7	88.35 ± 0.76	86.73 ± 3.84	79.15 ± 0.85	86.83 ± 1.14	35.94 ± 1.42
Focal Loss	γ = 2, α = 0.75	85.63 ± 0.64	85.31 ± 3.50	76.62 ± 0.90	88.02 ± 1.03	36.62 ± 1.65
BCE only	α = 1.00, β = 0	83.15 ± 0.49	81.41 ± 4.21	75.84 ± 1.15	89.27 ± 1.88	42.86 ± 1.91
Dice only	α = 0.00, β = 1.00	84.72 ± 0.75	86.36 ± 4.48	74.45 ± 0.94	83.42 ± 1.21	40.42 ± 1.73
BCE + Dice	α = 0.40, β = 0.60	87.96 ± 0.68	86.76 ± 3.75	80.05 ± 0.91	88.76 ± 1.08	35.73 ± 1.36
BCE + Dice	α = 0.45, β = 0.55	89.10 ± 0.87	87.04 ± 3.65	80.47 ± 0.85	89.41 ± 1.01	34.36 ± 1.27
BCE + Dice	α = 0.50, β = 0.50	90.04 ± 0.61	**88.10 ± 3.87**	80.82 ± 0.81	90.56 ± 1.43	33.27 ± 1.15
BCE + Dice	α = 0.55, β = 0.45	**90.47 ± 0.58**	87.83 ± 3.71	**81.26 ± 0.78**	**91.13 ± 0.95**	**32.94 ± 1.10**
BCE + Dice	α = 0.60, β = 0.40	88.87 ± 0.94	86.49 ± 3.88	80.35 ± 0.80	89.58 ± 0.97	34.98 ± 1.08

## Data Availability

The data used to support the findings of this study can be obtained from the corresponding authors upon request.

## Abbreviations

The following abbreviations are used in this manuscript:

ADCAM	Adaptive Dual-branch Channel Attention Module
CNN	Convolutional Neural Network
CT	Computed Tomography
GDUM	Gate-enhanced Dynamic Upsampling Module
HD95	95% Hausdorff Distance
ICH	Intracerebral Hemorrhage
IoU	Intersection over Union
MFEM	Multi-scale Feature Enhancement Module
ViT	Vision Transformer
